# Cost‐containment in hypofractionated radiation therapy: a literature review

**DOI:** 10.1002/jmrs.273

**Published:** 2018-03-13

**Authors:** Darren Hunter, Emily Mauldon, Nigel Anderson

**Affiliations:** ^1^ Department of Radiation Oncology Peter MacCallum Cancer Centre Melbourne Victoria Australia; ^2^ School of Medicine University of Tasmania Launceston Tasmania Australia

**Keywords:** Australia, cancer, cost, hypofractionation, radiotherapy

## Abstract

Recent technological advances in radiation therapy have allowed for greater accuracy in planning and treatment delivery. The development of hypofractionated radiation treatment regimens is an example, and has the potential to decrease the cost per episode of care, relative to conventional treatments. Our aim was to analyse published literature on the cost‐effectiveness and budgetary implications of hypofractionated radiation therapy. As such, this article will quantify the projected health care cost savings and address the optimal means of treatment delivery, associated patient outcomes, and implications arising from an increased use of hypofractionated regimens.

## Background

The financial stability of a public health care system is at the mercy of a number of conflicting factors. A dichotomy exists between those who view health care as expenditure (payers), and those who profit from it (providers). Similarly, the pressures of supply and demand challenge a dynamic health care system to be both proactive and responsive to change.^1^ Expenditure and cost‐control strategies must accommodate, and not exacerbate this tension. A multidimensional approach to cost‐containment is recommended to support an ever‐changing industry.

Cost‐containment strategies vary by country and geographic region. Stabile et al. 2 compared the various means of addressing rising health care costs in four high‐income nations – France, Germany, England and Canada; all of which are heavily reliant on public funding. Whilst significant differences exist in the organisational models of these health care systems, the cost‐containment strategies were comparable across nations. Three major approaches emerged; budget shifting, budget setting and direct/indirect controls. Budget shifting can simply be explained as limiting/omitting benefits, introducing user co‐payment and/or moving costs across different government platforms. In contrast, budget setting involves capping overall health care funding and/or adjusting the means of provider payment to activity‐based remuneration. The third means of cost‐containment, most influential at provider‐level, is by imposing direct or indirect controls. This includes greater Government control over price, infrastructure, technology and clinical practice guidelines. This in turn drives providers to seek more efficient and innovative means of achieving outcomes.

### The Australian context

In the 2014–15 financial year, the Australian Government invested $161.6 billion into the Australian health care system.^3^ This represents an increase of 2.8% over the previous financial year (2013–14 – $155 billion) and is indicative of the upward trend in health care spending (with an average annual growth of 4.6% over the last decade). In addition to the contributing factors of an ageing population and a developing health workforce, technological advancement accounts for a considerable cost‐driver within Australia. The investment in health care must be carefully balanced so as to support the expansion of service provision, but not succumb to financial penalty. Duckett & Willcox^1^ suggest that the control and/or reduction in health care cost has been integral to policy makers within the Australian Government over recent decades. Though this issue is not new, it continues to evolve with a health care system that grows in complexity.

In 2011, cancer accounted for the largest burden of disease amongst Australians.^4^ Conventional cancer treatment modalities include surgery, chemotherapy and radiation therapy. Radiation therapy is defined as the use of ionising radiation directed at a localised treatment site to kill and/or damage cancer cells.^4^ It may be used in conjunction with the aforementioned modalities, or as the primary treatment. Whilst there are a number of methods available for the delivery of radiation therapy, the most common application is external‐beam radiation therapy; by means of a linear accelerator (LINAC) machine.

Investment in radiation therapy occupies a substantial proportion of health expenditure. The Australian Government Department of Health 5 reports that in 2014–15, radiation therapy funding exceeded $411 million, of which $343 million constituted service delivery by Medicare benefits and a further $68 million comprised service improvement/expansion via the Radiation Oncology Health Programs Grant (ROHPG). Unlike the modest increase in overall health expenditure (2.8%), radiation therapy saw a net increase of 13.5% from the previous financial year. From 1988 to 2015, the number of LINACs installed nationally has increased considerably from 46 to 197 with a simultaneous rise in treatment facilities from 18 to 82 within the same period.^5^ The growing trend to establish more treatment facilities in regional and remote areas has enhanced access to radiation therapy services, but has come at a substantial cost. To offset capital gain investment in expanding service delivery, a number of cost‐containment strategies have been employed to increase value in service provision. These strategies include technological innovation, bolstered efficiency, public‐private partnership and service delivery targets.

A radiation therapy course is prescribed by a Radiation Oncologist and can constitute between 1 and 39 fractions (treatment sessions). In Australia, approximately 1.9 million fractions are delivered each year.^5^ The Australian Government provides significant funding, such that 80% of all services are charged at the Medicare Benefits Scheme schedule fee or less, thus most patients incur no (or very little) out‐of‐pocket expenses. Whilst 40% of patients elect for private treatment, Medicare provides part‐payment, with patients incurring the gap. 2014 fees for an average three‐field radiation therapy treatment across 20 fractions was $11,433. Of this amount, the Medicare rebate was $8784, with patients incurring the $2649 gap.

The major influencing factor in the escalation of radiation therapy course costs is indeed the fractionation schedule. A current clinical trial of hypofractionated prostate radiation therapy (PROFIT) has gained considerable media attention due to the strong likelihood that a shorter alternative to standard cancer treatment can elicit comparable outcomes for patients, at a considerably lower cost.^6^

Hypofractionation is a means of reducing the overall treatment course duration by delivering larger doses of radiation per fraction. Hegemann et al. 7 report that the first published studies on hypofractionated radiation therapy were conducted in Australia, Canada and the United Kingdom in the 1990s. The long distances travelled to access radiation therapy and the similarities of health care reimbursement across these three nations have fuelled an interest in more efficient means of treatment delivery without loss of quality and associated patient outcomes. The expansion of technology and innovation has fostered and reignited the use of hypofractionation as a safe and effective means of escalating dose to improve tumour kill at the treatment site. As such, variations of moderate (radical breast/prostate) and extreme (palliative Stereotactic Body Radiation Therapy or SBRT) hypofractionation are fast becoming established means of treatment delivery. Although hypofractionation presents a possible means of reducing financial costs, one must consider the additional resources and associated patient outcomes.

## Methodology

**Figure 1:**
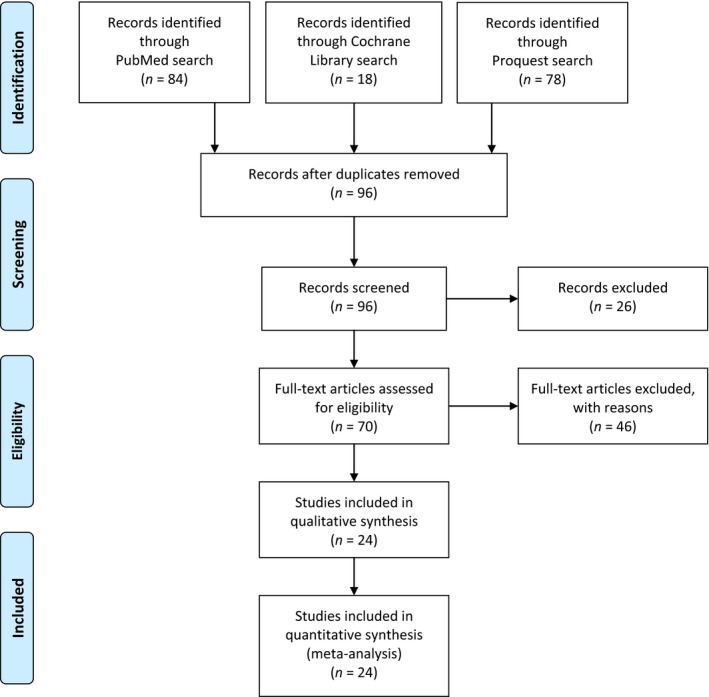
Literature search – screening and eligibility.

A literature review of PubMed, Proquest and the Cochrane Library was conducted in December 2017, using the search terms ‘hypofractionated’, ‘radiation’ and ‘cost’ (see Fig. 1). The combined search yielded a total of one‐hundred‐and‐eighty articles. In accordance with PRISMA guidelines,^8^ 84 articles were removed as duplicates, leaving 96 articles for consideration. A further 26 articles were removed with limitations placed on English language, human studies, full‐text and published within the past 10 years. The limitation placed on the past 10 years was selected so as to reflect the modern application of IMRT, VMAT and SBRT technologies that are cognizant within the current financial climate. Thus, seventy articles were assessed for eligibility; considerate of LINAC‐based delivery and relevance to cost‐analysis. Eligibility criteria removed a total of 46 articles – seventeen by evaluation of title and a further 29 removed by abstract. Twenty‐four articles were included for qualitative and quantitative analysis. Although the included articles varied in tumour stream and international context (see Table 1), commonalities existed in the discussion of health care cost reduction, optimal treatment delivery, patient costs and future considerations. These four emergent themes will be discussed below.

**Table 1:** Overview of literature reviewed

Reference	Tumour stream	Publication type	Recruitment figure	Key study outcome
Konski, AA,^9^ University of Pennsylvania, USA (2017)	Multiple	Review	N/A	Increased use of hypofractionation may increase patient value by reducing direct/indirect medical and productivity costs.
Laine, AM et al.,^10^ University of Texas Southwestern Medical Center, USA (2016)	Multiple	Review	N/A	Hypofractionation provides an efficacious, cost‐effective, efficient and convenient alternative to standard fractionation for a limited range of tumours.
Lievens, Y,^11^ University Hospital Gustuisberg, Belgium (2010)	Breast	Review	N/A	Despite acute gains to clinical outcomes and cost, long‐term data must assess the post‐treatment management costs attributed with increased hypofractionation.
Aneja, S et al.,^12^ Yale School of Medicine, USA (2012)	Prostate	Review	N/A	Hypofractionated prostate radiotherapy promises resource‐efficient and comparable patient outcomes to standard fractionation.
Kang, JJ et al.,^13^ David Geffen School of Medicine UCLA, USA (2015)	Bladder	Retrospective Analysis	26 patients (M/F), T2–4 N0–2M0 urothelial cancer	Hypofractionated partial bladder radiotherapy offers comparable local control and survival, with reduced cost/time.
Voong, KR,^14^ MD Anderson Cancer Centre, USA (2015)	Prostate	Conference Abstract	N/A	Hypofractionated prostate radiotherapy is seen to reduce costs, including the management of late radiation toxicities.
Greenup, RA et al.,^16^ Massachusetts General Hospital, USA (2017)	Breast	Retrospective Analysis	43,247 patients (F), T1–T2 N0 invasive breast cancer	Hypofractionated breast radiotherapy offers high‐value care, with a potential to reduce overall treatment costs by 39%.
Dwyer, P et al.,^17^ Princess Alexandra Hospital, Australia (2010)	Breast	Retrospective Analysis	279 patients (F), T1–T2 N0 invasive breast cancer	Hypofractionation could reduce costs by 24%; allowing for an additional 14 patients to be treated at the department each month.
Mortimer, JW et al.,^18^ University of New South Wales, Australia (2016)	Breast	Retrospective Analysis	196 patients (F). T1–T4 N0–3 invasive breast cancer	Hypofractionated breast cancer radiotherapy could reduce costs by 29.3% (22.1–32.0%).
Barry et al.,^19^ University of Louisville, USA (2015)	Breast	Cost‐effective Analysis	N/A	Conventional breast radiotherapy is financially burdensome in weeks 5–7, with no clear benefit to quality of care and patient outcomes. Once‐weekly hypofractionation could reduce costs by 65%.
Khan, AJ et al.,^20^ Robert Wood Johnson Medical School/Cancer Institute of New Jersey, USA (2016)	Breast	Retrospective Analysis	100 patients (F), T1–T4 N0–3 invasive breast cancer	Hypofractionation not only provides efficiency and cost saving, but translates to improved access to care in developing economies.
Konski, AA et al.,^21^ University of Pennsylvania, USA (2016)	Multiple	Cost‐effective Analysis	N/A	Increasing the rate of hypofractionation to 40% would reduce annual technical revenue by $540,661 and patient workflow by five patients (1–1.5 h) per day.
Muller‐Riemenschneider, FM et al.,^22^ Charite‐Universitatsmedizin, Germany (2009)	Cranial	Review	N/A	Increased use of hypofractionated techniques such as SRS must consider the economic implications of adaptive/dedicated equipment utilisation.
Deshmukh, AA et al.,^23^ University of Florida, USA (2017)	Breast	Cost‐effective Analysis	N/A	Hypofractionated breast radiotherapy offers the most cost‐effective option overall, as compared with conventional fractionation or brachytherapy (IORT).
Ojerholm, E et al.,^24^ University of Pennsylvania, USA (2017)	Breast	Editorial	N/A	Increased use of hypofractionated breast radiotherapy could provide equivalent patient outcomes, improve convenience and save $100 million each year.
Sharieff, W et al.,^25^ Juravinski Cancer Centre, Canada (2014)	Prostate	Cost‐effective Analysis	5000 patients (M), T < 2a, Gleason score <6, PSA <10 ng/mL prostate cancer	Providing equivalent efficacy and safety of SBRT delivery methods, arc‐based delivery offers the most cost‐effective method.
Zemplenyi, AT et al.,^26^ University of Pecs, Hungary (2016)	Prostate	Cost‐effective Analysis	N/A	Hypofractionated IMRT for prostate cancer offers the most cost‐effective means of delivery, as compared with standard fractionated 3D‐CRT or IMRT.
Lievens, Y et al.,^40^ Ghen University Hospital, Belgium (2015)	Lung	Cost‐effective Analysis	Financial data from 10 participating centres.	Time‐based reimbursement may provide a means of supporting the introduction of advanced radiotherapy techniques.
Min, C et al.,^27^ New York University, USA (2014)	Breast	Prospective Randomised Trial	84 patients (F), Tis‐T2, N0–Nx invasive breast cancer	At 3‐year follow‐up, hypofractionated breast radiotherapy appears to provide a promising option – particularly for elderly, non‐surgical candidates.
Bekelman, JE et al.,^28^ University of Pennsylvania, USA (2014)	Breast	Retrospective Analysis	Financial data from 14 commercial health care plans.	Though the rate of use has increased, a mere 21.2–34.5% of eligible patients receive hypofractionated breast radiotherapy.
Ohri, N & Haffty, BG,^30^ Rutgers Cancer Institute of New Jersey, USA (2018)	Breast	Review	N/A	Hypofractionated approaches to breast cancer (including accelerated partial breast irradiation) offer an effective alternative for selected patients.
Voong, KR et al.,^35^ The John Hopkins School of Medicine, USA (2017)	Prostate	Prospective Randomised Trial	204 patients (M), T1–T2, Gleason score 6–8	Moderate hypofractionated prostate radiotherapy offers increased value, considerate of late toxicity.
Eblan, MJ et al.,^36^ University of Pennsylvania, USA (2014)	Breast	Review	N/A	Hypofractionated breast radiotherapy offers cost savings, increased patient throughput and reduced waiting lists.
Konski, AA,^38^ Wayne State University, USA (2012)	Prostate	Commentary	N/A	Although hypofractionated prostate radiotherapy offers convenience to patients, brachytherapy provides further cost reduction.

## Discussion

### Health care cost reduction

Current research suggests that the number of US patients requiring radiation therapy is due to rise from 470,000 in 2010 to 575,000 by 2020.^9^ This growth in patient numbers needs to be met with more cost‐effective means of treatment delivery. It is important to acknowledge that radiation therapy is largely considered cost‐effective by means of Incremental Cost‐Effectiveness Ratio (ICER) analyses; certainly in comparison with systemic treatments.^10^ However, within the period of 1996 to 2016 there has been a considerable increase in cost‐effective analysis publications within the context of radiation therapy practice. Amongst the various components that are thought to contribute to cost‐effectiveness, changes to fractionation have accounted for the second largest body of work in this field. Cost‐calculation models have demonstrated that daily operating expenditures outweigh capital machine costs in the planning and delivery of radiation therapy treatment.^11^ As such, Aneja et al.^12^ suggests that radiation therapy costs are largely a product of time; given by the duration of each fraction multiplied by the total number of fractions. Therefore, hypofractionation has the potential to reduce the burden of rising health care costs in the area of radiation therapy.^11^, ^13^ It is estimated that the contribution of direct planning and treatment delivery costs are 20% and 80% respectively.^13^ The use of hypofractionation is most commonly discussed in prostate and breast applications, of which constitute the two largest patient cohorts.^4^

Prostate cancer accounts for the longest course of radiation therapy by comparison with other anatomical sites, due to the radioresistant nature of prostatic tissue. International standards suggest that prostate radiation therapy is given as 75.6–81.0 Gy in 1.8–2.0 Gy per fraction over 7–9 weeks.^12^, ^14^ In Australia, typical doses are either 74 Gy (in 37 fractions) or 78 Gy (in 39 fractions) in the setting of post‐prostatectomy or intact prostate respectively. In either case, the treatment is rather cumbersome and very expensive. US data suggests that a typical course of conventional prostate radiation therapy can cost in the vicinity of $30,241–$37,125 accounting for PSA testing, imaging, symptom management and consultation fees.^12^, ^14^ In contrast, a moderate hypofractionation schedule of 30 fractions could see a saving of approximately $7000 per patient, with the course cost weighing in at $22,957.^14^ With an estimated 180,000 new diagnoses of prostate cancer in the United States alone each year, this could translate to significant health care cost savings.^12^

The Australian Medicare Benefits Schedule (MBS) provides a clear framework for the billing of all radiation therapy procedures, including CT/simulation, dosimetry, image verification and daily treatment provision. MBS item codes vary with the level of complexity of treatment and planning processes.^15^ As such, the most advanced form of treatment planning and delivery (Intensity Modulated Radiation Therapy – IMRT), incurs the greatest reimbursement. Thus, it is highly advantageous for centres to implement IMRT planning, with additional benefits to patient outcomes and centre revenue. IMRT planning incurs a higher rate of reimbursement of CT and dosimetry at $710.55 and $3313.85 respectively. In contrast, conventional means of planning would be charged at a rate of $658.60 for CT and $1120.75 for dosimetry.

By stark contrast, the daily treatment costs are not significantly different and there is no financial incentive to deliver fewer treatments. Irrespective of the duration of treatment, IMRT treatment is billed at $182.90 per treatment, as opposed to $211.45 for a 5‐field conformal technique. In either case, a hypofractionated course would simply suffer a considerable net loss of income. Inclusive of equal planning costs, a proposed hypofractionated prostate IMRT schedule (20 fractions) would cost $7682.40, compared with $11,157.50 for standard fractionation (39 fractions).

Breast radiation therapy has similarly been the discussion of debate for hypofractionation in recent history. Greenup et al. 16 suggests that breast cancer treatment costs are higher than any other tumour stream, with an estimated cost of $20 billion by 2020 in the United States. Radiation therapy plays an integral part of standard breast cancer treatment, as such, contributing heavily to this cost burden.^17^ Conventional radiation therapy courses for breast cancer are provided as 50 Gy in 25 fractions. A growing trend to hypofractionation has seen a number of patients receive 40 Gy in 15 fractions, or 42.5 Gy in 16 fractions. While comparatively smaller than the prostate hypofractionated regime, a reduction in nine fractions has seen a reduction in cost per patient from $13,358 to $8328 in the United States – a staggering difference of 38%.^16^ Australian data has echoed a comparable 32% cost reduction from $8272 to $5613.^18^ These figures consider not only the additional costs per fraction delivered, but the associated weekly management costs – of which are substantially burdensome beyond week 4 of breast cancer treatment.^19^

Research conducted in the USA found that 57% patients underwent treatment regimens for breast cancer that were unnecessarily costly, accounting for a total $420.2 million in 2011.^16^ Had patients been treated with more fiscally conservative radiation therapy options, this figure would have been reduced by $164.0 million to $256.2 million – a net cost saving of 39%. Extrapolation of similar Australian data suggests a comparable reduction in expenditure for breast radiation therapy from $31.3 million to $22.2 million – accounting for a $9.1 million saving (29% reduction).^18^ Khan et al.^20^ suggest that these savings could be redirected, resulting in profound additional benefits in treatment access via a reduction in waiting times and improved service delivery – particularly in emerging economies of the world.

### Optimal treatment delivery

There is consensus in the literature to suggest a growing trend to the use of hypofractionated regimens. The further development and implementation of sophisticated treatment planning systems and image verification has allowed for improved accuracy of treatment, in turn permitting the use of hypofractionation in a safe manner.^9^ In 2010, 64% of radiation oncologists in the United States had access to equipment sufficient for the application of hypofractionation techniques. Research suggests that uptake of hypofractionation across the United States in 2011 was observed in 8% of prostate cancer patients, 44% of inoperable lung cancer patients and 20–35% in the setting of breast cancer.^16^, ^21^ In contrast, the uptake figures across Canadian breast cancer patients is currently estimated at 70%; perhaps reflecting differences in practice attributed to varying models of health care.

Aneja et al. 12 propose that radiation therapy costs are proportionate to the number of fractions. While there is truth to this statement, the escalation of costs is not so simply derived by the number of treatments, but also the means of treatment delivery. Significant variations in cost‐per‐fraction exist between moderate and extreme hypofractionation (stereotactic body radiation therapy – SBRT). Similarly, a range of treatment techniques can be applied including external beam radiation therapy or brachytherapy, resulting in a different cost per fraction.^11^ Variations on the delivery of external beam radiation therapy (arc, fixed gantry and robotic) can also alter treatment costs. Economic efficiency must also consider both the downstream costs and access to systems – dedicated or adaptive – to provide hypofractionated radiation therapy.^10^, ^22^, ^23^, ^24^

Prostate cancer is subject to a range of options of conventional, moderate and extreme hypofractionation. As such, there are currently no clear guidelines for clinicians in implementing prostate hypofractionation. Aneja et al. suggest this may contribute to lower rates of uptake for prostate cancer, compared with breast and lung cancer.^12^ Moderate hypofractionation aims to maintain tumour control and toxicity within a shorter fractionation, while extreme hypofractionation provides a more aggressive approach of higher dose in fewer fractions (but is subject to uncertainty surrounding the implementation and outcomes). Sharieff et al. 25 found that extreme hypofractionation for prostate cancer can reduce treatment costs by approximately 27–55%, compared with conventional treatment. Similarly, moderate hypofractionation accounts for a 22% decrease in cost, as opposed to conventional treatment.^26^ Furthermore, arc‐based delivery is considered the most cost‐effective treatment, allowing for a 21–32% reduction in cost in comparison with equivalent applications of robotic or fixed‐gantry radiation therapy.^25^

Extreme hypofractionation is gaining acceptance as a standard modality in lung cancer. However, research has demonstrated that the treatment costs for extreme hypofractionation are similar to conventional treatment, whereas moderate hypofractionation is less costly.^26^ This disparity is attributed to a number of factors such as additional time (planning and daily treatment) and resources (senior radiation oncologists, physicists, and skilled radiation therapists). The varying costs of extreme hypofractionation reported may also reflect local differences in service delivery, such as departmental experience with the use of these technologies.

### Patient costs

Whilst evidence supports the cost‐saving attributed with hypofractionation, a paramount concern is, of course, patient outcomes associated with treatment. The value of radiation therapy is determined by patient satisfaction and, similarly, treatment‐related outcomes. It is largely accepted that late toxicity is strongly correlated with the dose per fraction – a key component of hypofractionation.^12^ However, research suggests that the toxicity attributed to hypofractionation is largely comparable with that seen in conventional radiation therapy courses.^9^

Hypofractionation in breast cancer is seen to provide promising local control rates and good cosmetic outcomes.^16^, ^27^, ^28^ Published data from the UK START trials confirm the safety and efficacy of hypofractionated breast radiation therapy,^29^ negating prior concerns of long‐term (cardiac) post‐treatment management.^11^ The combined START‐A and START‐B trials enrolled a total of 4451 women across the UK between 1999 and 2002. Analysis at 10‐year post‐treatment has confirmed no long‐term detriment with the use of hypofractionation, but rather comparative patient outcomes for START‐A, and rather, improved rates of disease‐free survival and overall survival with the START‐B hypofractionated intervention (40 Gy in 15 fractions). Khan et al. 20 proposes that the equivalence of conventional and hypofractionated radiation therapy for breast cancer is now undisputed. As such, hypofractionation in breast cancer is supported by current practice guidelines and the Choosing Wisely initiative endorsed by the American Society for Radiation Oncology.^24^, ^30^

There is considerable evidence to support the use of hypofractionation in the setting of lung cancer and palliation. Extreme hypofractionated regimens for lung cancer offer favourable outcomes with a cost‐effective and less invasive procedure than surgery and associated hospitalisation.^21^ Furthermore, multiple studies across Europe and the USA have demonstrated an equivalent outcome in hypofractionated palliation compared with conventional approaches – though this technique is heavily underutilised.^21^, ^31^, ^32^, ^33^

Hypofractionation in prostate cancer is subject to debate surrounding clinical efficacy and increased bowel/urinary toxicity. Recent literature has demonstrated a significant increase in gastrointestinal and genitourinary toxicity across Australian and Canadian demographics.^12^, ^34^ However, on cost alone, symptom management represents a small part (<10%) of the average total cost for patients who develop grade 2/3 bowel or urinary toxicity.^13^ In contrast, additional studies have demonstrated that hypofractionation is effective in the management of prostate cancer, with attributed health gains.^21^, ^26^, ^35^

Hypofractionated radiation therapy presents patients with a number of attractive benefits. Reduced waiting lists and resultant increased capacity for radiation therapy access is a striking benefit of hypofractionation.^17^, ^36^ Current data predict considerable insufficiencies with service provision to meet the demands of 52% of new cancer diagnoses by 2022.^37^ Furthermore, this treatment regimen provides a convenience to patients, with fewer commutes to the radiation therapy centre.^12^, ^36^, ^38^ As such, there are potential reductions in patient out‐of‐pocket expenses including the cost of commuting/parking, and income/productivity loss associated with longer treatment courses.^11^, ^25^, ^28^, ^38^ Further savings to care provision may be associated with a reduced need for patient accommodation, nursing/doctor consultations and the State‐funded Patient Assisted Transport Schemes.^39^ Therefore, the use of hypofractionated radiation therapy may provide a fiscally desirable alternative not only to conventional radiation therapy, but also to surgical interventions such as prostatectomy or mastectomy.^9^

This modern approach to treatment is yet to constitute standard of care in many centres worldwide. In the light of ongoing uncertainty surrounding some applications of hypofractionation, further challenges exist in gaining acceptance amongst radiation oncologists, who may hold firm beliefs on conventional treatments.^21^, ^28^ Ultimately, further research is required to quantify the value of hypofractionation in achieving better clinical outcomes and enhanced quality of life across a broad range of treatment applications.^9^

### Future considerations

The expansion of hypofractionated radiation therapy must take into account the direct impacts on service providers, the health workforce and advances in clinical practice and technological development. A reduction in Government funding for radiation therapy treatments will result in a decline in hospital department revenue.^17^ Evidence suggests that a typical US radiation therapy department could see a reduction in technical revenue by $540,661 should hypofractionation constitute 40% of the patient load.^21^ Given that lung, prostate, breast and palliation account for a majority of sites treated with radiation therapy, this figure is not unrealistic. In addition to revenue loss, this hypothetical shift would see a reduced workflow of approximately five patients (or 1.5 h) per day, unless counterbalanced by an increase in billable treatment and planning sessions.^17^

With a net loss of revenue, departments will likely be challenged to address budget shortfalls at a local level.^38^ With a reduction in the need to expand the fleet of linear accelerators, equipment funding (i.e. ROHPG) could be redirected to departments as a means of ensuring research and development activities progress.^21^ Alternatively there may be a push back from department level to evaluate the reimbursement model in an attempt to regain a comparable revenue stream.^17^, ^30^ In the United States, there is currently movement afoot to adjust the current fee‐for‐service model to a bundle payment for care improvement (BPCI) as part of revised Affordable Care Act (ACA).^19^ However, Konski et al. 21 warns that ‘what is not known is whether the transition from standard therapy to more technically demanding but higher‐reimbursed therapy will offset the loss of volume’ (p. e581).

Reducing revenue will likely see impacts on the workforce.^38^ Research suggests that the increase in hypofractionation will likely exacerbate a projected oversupply of radiation oncologists from 2015 to 2025.^9^ Workforce reduction would likely cause significant issues across rural and remote treatment centres, in particular. Staff training and education would need to evolve with the changing landscape. Konski et al. 21 argues that the effects of hypofractionation could fuel a reduction in student numbers across the medical radiation programs. Reflective of the current climate, the 2014 American Society of Radiologic Technologists enrolment report indicated that 18.8% of all medical radiation programs will likely decrease student enrolment, and similarly, there was an observed 1.6% decrease (from 2013 to 2014) in the success of students gaining employment 6‐month post‐graduation. One might suggest that further reduction in the workforce will only fuel this decline.

## Conclusion

Hypofractionation provides a feasible means of reducing health expenditure in the setting of radiation therapy. A large number of radiation therapy centres across Australia now have the requisite equipment and staff experienced in this mode of treatment delivery, aiding transition and implementation at a department level. Evidence supports the potential for significant cost‐saving across a health care sector that is projected to increase and impose a significant burden on the overall Australian health care budget.

Whilst hypofractionated radiation therapy could constitute an effective cost‐containment strategy at Government level, one must consider the wider implications. Further research and cost‐effectiveness analyses are required to ascertain governance around the best method of delivery. A number of techniques are employed worldwide, with varying success. The best value in the context of the Australian Medicare Benefits Scheme must be analysed and considered with projected workforce growth and patient demand. Patient outcomes must be paramount to this research, such that treatment efficacy is maintained and/or improved. Additional costs including long‐term side‐effect management must be considered, so as not to impart further burden on another health care sector.

The growth of the profession must also be considered. Funding must continue to drive innovation and development in a field that is highly dependent on progressive technology. The impact of increased hypofractionated delivery must coincide with reducing the strain of waiting lists and improved access to services. Similarly, the intake of students into educational programs must align with the projected clinical services so as to avoid over‐supply and unemployment within this health care sector.

Ultimately, hypofractionation presents a promising approach to cost‐containment in radiation therapy and the wider health care budget, but the issues of implementation and potential workforce repercussions must be addressed and planned for.

## Conflict of Interest

The authors declare no conflict of interest.
